# Detection of porcine epidemic diarrhea virus (PEDV) IgG and IgA in muscle tissue exudate (“meat juice”) specimens

**DOI:** 10.1186/s40813-018-0107-4

**Published:** 2018-12-18

**Authors:** Korakrit Poonsuk, Ting-Yu Cheng, Ju Ji, Jeffrey Zimmerman, Luis Giménez-Lirola

**Affiliations:** 1 Department of Veterinary Diagnostic and Production Animal Medicine, College of Veterinary Medicine, Iowa State University, 2203 Lloyd Veterinary Medical Center, Iowa, 50011-1134 USA; 2 Department of Statistics, College of Liberal Arts and Sciences, Iowa State University, 2438 Osborn Drive, Ames, Iowa, 50011-1090 USA

**Keywords:** Porcine epidemic diarrhea virus; meat juice, Muscle exudate, Antibody, Enzyme linked immunosorbent assay

## Abstract

The diagnostic performance of porcine epidemic diarrhea virus (PEDV) IgG and IgA ELISAs was evaluated using paired serum and meat juice samples collected from PEDV-negative (*n* = 50) and PEDV-inoculated pigs (*n* = 87). Serum samples were tested by PEDV (IgG, IgA) ELISAs using a procedure performed routinely at the Iowa State University-Veterinary Diagnostic Laboratory (ISU-VDL). Serum samples were tested using PEDV serum IgG and IgA ELISA procedures as routinely performed at the Iowa State University-Veterinary Diagnostic Laboratory (ISU-VDL). Serum samples were diluted 1:50 and conjugate concentrations were 1/20,000 for IgG and 1/3000 for IgA. Meat juice samples were tested using the serum PEDV IgG and IgA ELISAs, with modifications, i.e., meat juice samples were diluted 1:25 and conjugate concentrations were 1/40,000 for IgG and 1/10,000 for IgA. Receiver operator characteristic (ROC) curve analyses were used to estimate diagnostic sensitivities and specificities over a range of sample-to-positive (S/P) cutoffs. Consistent with previous reports, this study showed that the PEDV IgG and IgA meat juice ELISAs provided excellent diagnostic performance and suggest that meat juice recovered from samples collected at slaughter could be used in routine PEDV surveillance.

## Background

“Meat juice”, the transudate produced as frozen muscle tissue undergoes the process of thawing, is composed of intracellular fluid, extracellular fluid, blood, and lymph [1]. As such, meat juice contains antibodies, albeit at a lower concentration than serum [1], and diagnostically sensitive and specific meat juice antibody ELISAs have been described for porcine reproductive and respiratory syndrome virus (PRRSV), influenza A virus, *Mycoplasma hyopneumoniae*, *Salmonella* spp., *Trichinella* spp., *Yersinia enterocolitica*, and *Toxoplasma gondii* [2]. Meat juice is particularly compatible with abattoir-based surveillance because muscle tissue samples are easily collected at slaughter [2, 3]. Typically, meat samples collected in the abattoir are hard-frozen and then thawed after arrival at the laboratory to produce the specimen. Samples can be stored frozen or tested immediately. Abattoir-based meat juice surveillance or monitoring has been described for salmonella, *Toxoplasma gondii*, and PRRSV [2, 4]. Therefore, the objective of this study was to evaluate the detection of porcine epidemic diarrhea virus (PEDV)-specific IgG and IgA in individual serum and meat samples (*longissimus dorsi* muscle).

## Methods

The detection of PEDV-specific IgG and IgA in individual specimens was evaluated using serum and meat samples from PEDV naïve (*n* = 50) and PEDV inoculated pigs (*n* = 87) at 14 days post-inoculation. PEDV positive pigs were orally inoculated with 1 × 10^3^ TCID_50_ cell-culture propagated PEDV (isolate USA/IN/2013/19338E) at 4 or 2 days of age. All piglets were closely observed for clinical signs through day post-inoculation (DPI) 14 or until humane euthanasia was necessary. Productive infection was confirmed by positive PEDV RT-rtPCR results on individual fecal samples collected ≥4 days post inoculation and tested at the Iowa State University Veterinary Diagnostic Laboratory [5]. PEDV negative specimens were collected from 21 piglets from PEDV naïve sows farrowed in a biosafety level-2 (BSL-2) research facility and 29 finishing pigs originating from a PEDV-free commercial swine farm. All animals were housed in research facilities accredited by the Association for Assessment and Accreditation of Laboratory Animal Care (AAALAC). All animal procedures were conducted with the approval of the Iowa State University Office for Responsible Research.

Serum and meat (*longissimus dorsi* muscle tissue) samples were collected from all pigs at necropsy. Meat juice samples were harvested from the muscle tissue after one freeze-thaw cycle. Serum and meat juice samples were randomly ordered and tested using PEDV ELISAs (IgG and IgA), as fully described elsewhere [6]. In brief, PEDV ELISA plates were coated with purified PEDV (USA/NC35140/2013) viral particles propagated in Vero cells. To prepare the antigen, propagated PEDV was subjected to one freeze-thaw cycle (− 80 °C). The harvested material were centrifuged at 4000 x *g* for 15 min to remove cell debris. The virus pellet was harvested and washed (2X) with PBS (1X, pH 7.4). The purified virus was resuspended in PBS (1X pH 7.4) at a dilution of 1:100 of the original supernatant volume and stored at − 80 °C. The virus was coated onto polystyrene 96-well microtitration plates (Nalge Nunc Corp.). PEDV serum and meat juice ELISA (IgA, IgG) positive and negative controls consisted of samples controls collected from pigs of known PEDV status. Positive and negative controls were included in duplicate in every plate. To perform the test, 100 μl of diluted (1,50) serum was aliquoted into each well and the ELISA plate incubated for 1 h at 25 °C. For serum, 1:20,000 peroxidase-conjugated goat anti-pig IgG (Fc) antibody (Bethyl Laboratories Inc., Montgomery, TX) or 1:3000 goat anti-pig IgA (Bethyl Laboratories Inc.) was added to each well and the plate incubated at 25 °C for 1 h. The reaction was visualized by adding 100 μl of substrate solution (TMB, Dako North America, Inc., Carpinteria, CA) to each well, then incubating the plate at 25 °C for 5 min. The reaction was stopped by the addition of 50 μl of stop solution (1 M sulfuric acid). Reactions were measured as optical density at 450 nm using an ELISA plate reader (Biotek® Instruments Inc., Winooski, VT). The results were represented as sample-to-positive (S/P) ratios:

$$ S/P\  ratio=\frac{\left( sample\  OD- blank\ well\ control\ mean\  OD\right)\ }{\left( positive\ control\ mean\  OD- blank\ well\ control\ mean\  OD\right)} $$

Meat juice samples were tested using the protocol described above, except that samples were diluted 1:25 and conjugate concentrations were 1/40,000 and 1/10,000 for IgG and IgA, respectively.

Serum and meat juice ELISA S/P data were analyzed using commercial statistical software (SAS® 9.4, SAS® Institute Inc., Cary NC, USA). Receiver-operator characteristic (ROC) curve analyses were used to estimate the cutoff thresholds and associated diagnostic sensitivities and specificities for both serum and meat juice IgG and IgA ELISAs. Confidence intervals for diagnostic sensitivities and specificities were calculated using the exact Binomial formula. The strength of the association between individual pig serum and meat juice IgG and IgA ELISA S/P responses was evaluated by linear regression analysis.

## Results and discussion

**Fig. 1:**
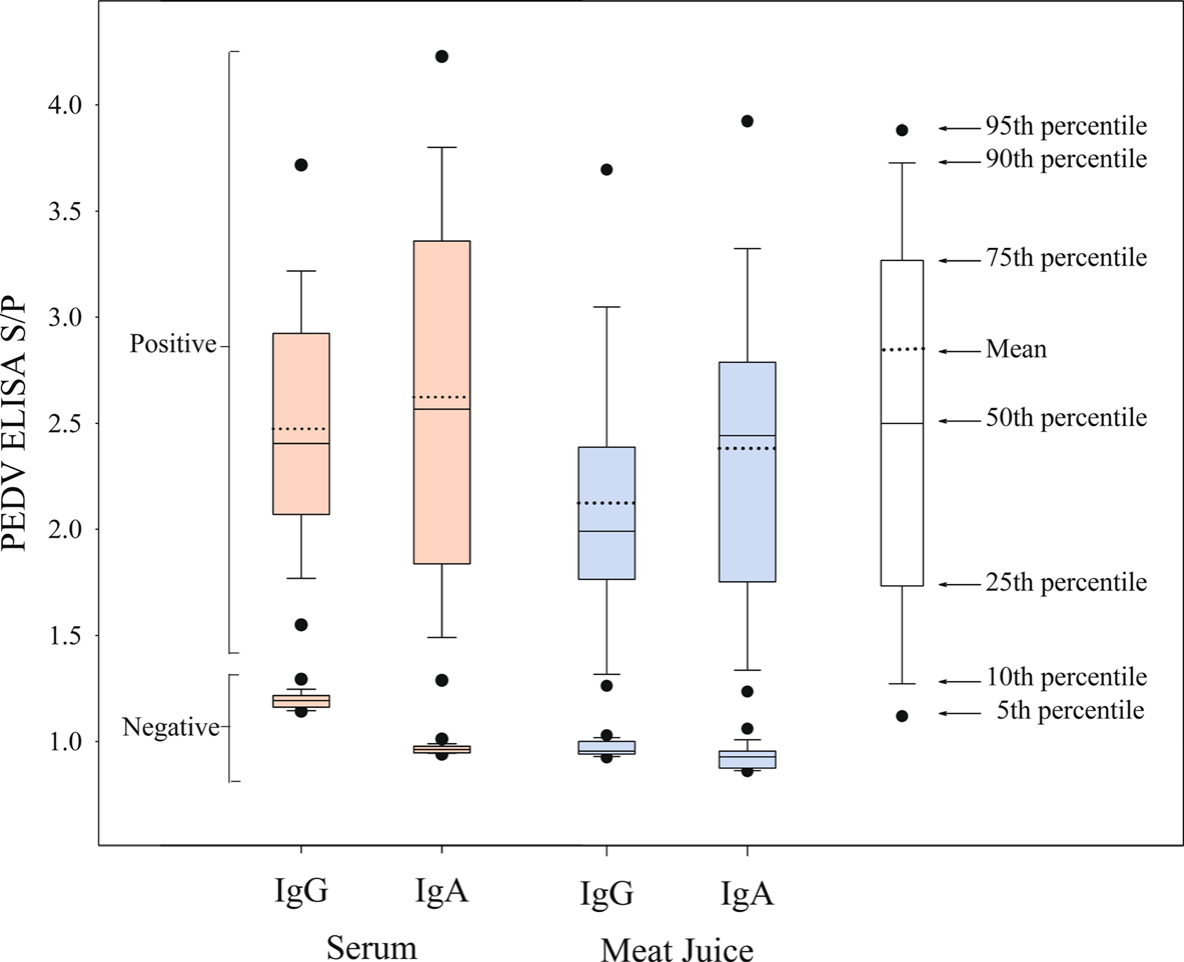
Distribution of PEDV IgG and IgA ELISA S/*P* values based on testing serum and meat juice samples from PEDV-negative (*n* = 50) and PEDV-inoculated pigs (*n* = 87)

**Fig. 2:**
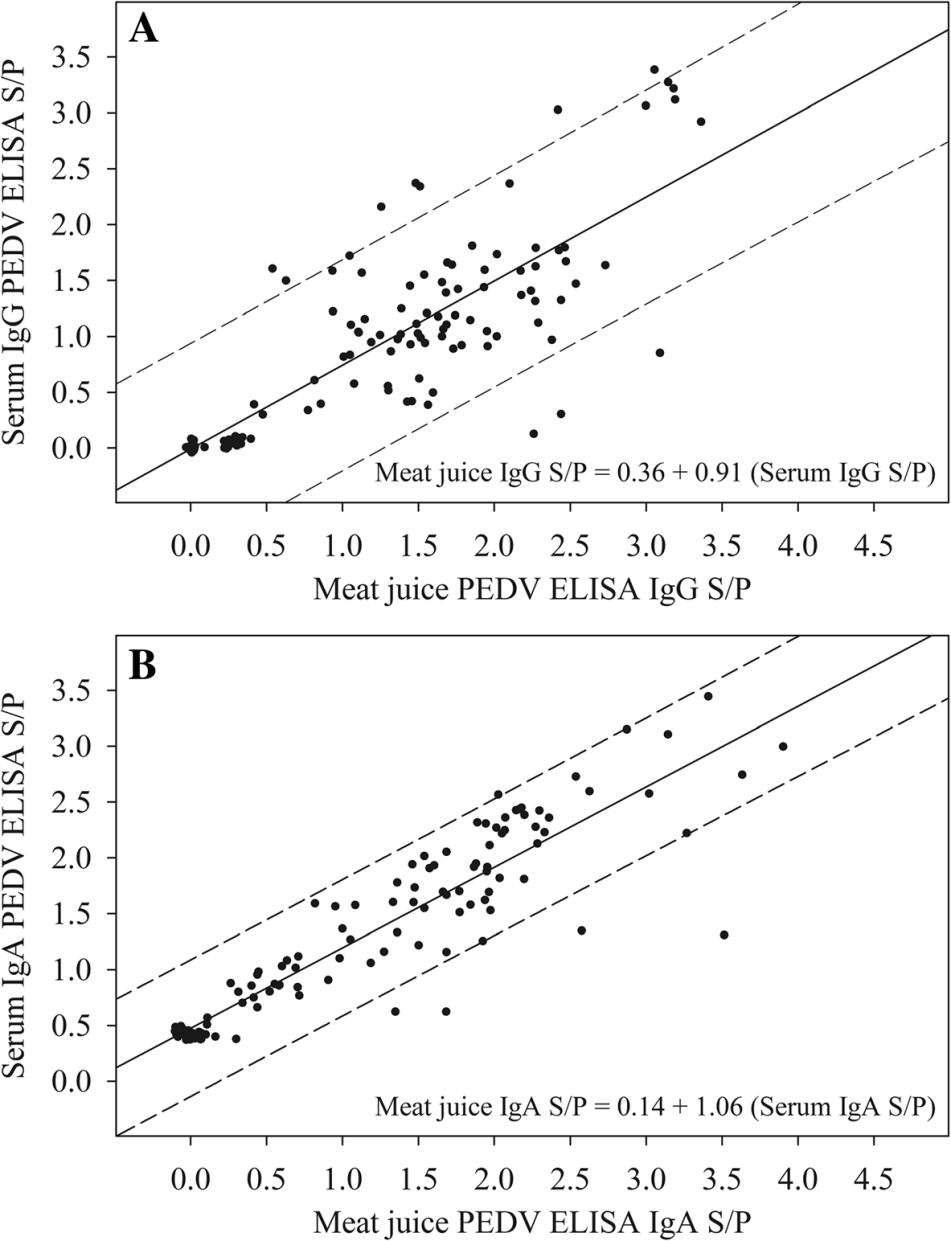
The association between serum and meat juice S/P results was significant for both the PEDV IgG (**a**) and IgA (**b**) ELISAs (linear regression; *p* < 0.0001)

All pigs in the PEDV-inoculated group were clinically normal in appearance and behavior prior to the inoculation. Some pigs showed watery diarrhea during the observation period and all fecal samples were PEDV-specific rRT-PCR positive within 4 days post-inoculation. The distribution of PEDV ELISA S/P results for serum and meat juice (Fig. 1) showed that the IgA and IgG serum and meat juice ELISAs provided clear discrimination between positive and negative samples. The association between serum and meat juice S/P results (Fig. 2) was significant for both the PEDV IgG (R^2^ 0.64) and IgA (R^2^ 0.89) ELISAs (linear regression; *p* < 0.0001). Test performance based on ROC analyses of serum and meat juice ELISA results are reported in Table 1 over a range of cutoffs. These data are consistent with previous reports. That is, detectable levels of antibodies against a variety of pathogens have been reported in meat juice, e.g., pseudorabies virus [7], PRRSV [4], classical swine fever virus [8], porcine circovirus type 2 [9] and others. A previous study using specimens from 49 swine herds in Germany reported that antibodies against *Salmonella* spp., *Trichinella* spp., *Yersinia enterocolitica*, *Toxoplasma gondii*, *Mycoplasma hyopneumoniae*, influenza A virus (H1N1 and H3N2), and PRRSV could be detected in meat juice [2].

**Table 1:** Porcine epidemic diarrhea virus (PEDV) serum and meat juice IgG and IgA ELISA diagnostic sensitivity (Se) and specificity (Sp) by cutoff. Estimates based on pig-matched serum and meat juice samples (*n* = 137 pigs)

	Cutoff (S/P)^a^	PEDV IgG ELISA		PEDV IgA ELISA	
		Se (95% CI)	Sp (95% CI)	Se (95% CI)	Sp (95% CI)
Serum	0.1	100 (95.8, 100)	56.0 (41.3, 70.0)	100 (95.8, 100)	72.0 (57.5, 83.8)
	0.2	100 (95.8, 100)	58.0 (43.2, 71.8)	100 (95.8, 100)	100 (92.9, 100)
	0.3	100 (95.8, 100)	86.0 (73.3, 94.2)	98.9 (93.8, 100)	100 (83.9, 100)
	0.4	100 (95.8, 100)	100 (92.9, 100)	95.4 (88.6, 98.7)	100 (83.9, 100)
	0.5	98.9 (93.8, 100)	100 (92.9, 100)	94.3 (87.1, 98.1)	100 (83.9, 100)
	0.6	96.6 (90.3, 99.3)	100 (83.9, 100)	90.8 (82.7, 95.9)	100 (83.9, 100)
Meat juice	0.1	100 (95.8, 100)	98.0 (89.4, 99.9)	100 (95.8, 100)	94.0 (83.5, 98.7)
	0.2	100 (95.8, 100)	100 (92.9, 100)	97.7 (91.9, 99.7)	96.0 (86.3, 99.5)
	0.3	98.9 (93.8, 100)	100 (92.9, 100)	96.6 (90.3, 99.3)	100 (92.9, 100)
	0.4	93.1 (85.6, 97.4)	100 (92.9, 100)	94.3 (87.1, 98.1)	100 (92.9, 100)
	0.5	89.7 (81.3, 95.2)	100 (92.9, 100)	88.5 (79.9, 94.3)	100 (92.9, 100)
	0.6	85.1 (75.8, 91.8)	100 (92.9, 100)	83.9 (74.5, 90.9)	100 (92.9, 100)

^a^Sample-to-positive (S/P) = (sample OD – blank well control mean OD)/(positive control mean OD – blank well control mean OD)

Porcine coronaviruses, particularly PEDV, transmissible gastroenteritis virus, and porcine deltacoronavirus, have caused devastating losses at the global level [10]. New swine coronaviruses, e.g., swine acute diarrhea virus (SADS-CoV), continue to emerge with effects yet to be determined [11]. Highly transmissible and stable in the environment [10], these viruses have moved rapidly across pork-producing regions of the world. The exception to the rule, Canada has been effective in slowing the spread of PEDV and PDCoV and may ultimately succeed in eliminating both viruses [12]. The Canadian effort is based on extensive surveillance of animals, farms, and the production chain, i.e., livestock assembly yards, abattoirs, truck wash stations, and livestock trailers, by RT-rtPCR. If control and elimination is successful, other regions may choose to follow suit. However, swine coronaviruses will continue to circulate widely in swine populations in much of the world. Thus, inexpensive but effective surveillance methods will be needed to detect incursions, guide program responses, and maintain freedom from these pathogens.

In the context of surveillance, meat juice-based testing presents significant advantages. Porcine muscle specimens are readily collected at the abattoir and avoid the biosecurity risks inherent to on-farm visits. The disadvantage of this approach is that infection is only recognized retroactively. Regardless, meat juice is easily recovered by thawing the frozen meat sample and is compatible with high-throughput testing.

Previous reports showed that the kinetics of ELISA-detectable PEDV IgG and IgA differed among specimen types, e.g. serum, oral fluids, and mammary secretions [6, 13]. Since PEDV infection occurs in the gut and stimulates mucosal immunity, IgA is usually higher than IgG in oral fluids and mammary secretions, but lower than IgG in serum [14]. For completeness, both IgG and IgA antibody ELISAs were evaluated for their ability to discriminate between PEDV-negatives and -positives in the meat juice matrix. Although the duration of detectable PEDV antibodies in meat juice specimens has not been described, Bjustrom-Kraft et al. (2016) reported that PEDV-specific IgG and IgA was detected in serum and oral fluid specimens for at least 4 months post-inoculation using the same PEDV IgG and IgA ELISAs as performed in this study [13]. This suggests that meat juice specimens collected from PEDV-positive populations at the abattoir will also contain ELISA-detectable levels of antibody.

Test performance of the PEDV IgG and IgA ELISAs was previously described [13]. In this study, even though the broad test diagnostic sensitivity and specificity were reported due to number of the animals, these ELISAs were found to be highly diagnostically specific and sufficiently diagnostically sensitive at the individual pig level to be useful in surveillance. The results of this study suggest that a cutoff of S/*P* ≥ 0.5 would be appropriate for surveillance using either the IgG or IgA meat juice ELISAs (Table 1). This cutoff intentionally maximizes diagnostic specificity at the cost of sensitivity because false positive results quickly undermine confidence in the surveillance program. The shortcoming of a lower probability of detection (diagnostic sensitivity of ~ 90%) is offset by the fact that most individuals in PEDV-positive populations are infected. Furthermore, the availability of both IgG and IgA PEDV meat juice ELISAs would provide for screening and confirmatory assays based on different antibody isotypes. Given these advantages, this approach should be given consideration if the industry elects to intensify efforts to monitor and/or control PEDV.

## Abbreviations

ELISA: Enzyme-linked immunosorbent assay
IgA: Immunoglobulin A
IgG: Immunoglobulin G
OD: Optical density
PBS: Phosphate buffer saline
PDCoV: Porcine delta coronavirus
PED/PEDV: Porcine epidemic diarrhea/ PED Virus
PRRS/PRRSV: Porcine reproductive and respiratory syndrome/PRRS virus
ROC: Receiver operator characteristic curve analysis
RT-rtPCR: Real-time reverse transcription polymerase chain reaction
S/P: Sample-to-positive ratio
SADS-CoV: Swine acute diarrhea virus
